# Extensive testing of a multi-locus sequence typing scheme for *Giardia duodenalis* assemblage A confirms its good discriminatory power

**DOI:** 10.1186/s13071-022-05615-x

**Published:** 2022-12-26

**Authors:** Christian Klotz, Anna Rosa Sannella, Filip Weisz, Umer Chaudhry, Jacek Sroka, Pavla Tůmová, Eva Nohýnková, Ralf Ignatius, Toni Aebischer, Martha Betson, Karin Troell, Simone M. Cacciò

**Affiliations:** 1 Department of Infectious Diseases, Unit for Mycotic and Parasitic Agents and Mycobacteria, Robert Koch Institute, Berlin, Germany; 2 Department of Infectious Diseases, Istituto Superiore Di Sanità, Rome, Italy; 3 Institute of Immunology and Microbiology, First Faculty of Medicine, Charles University, Prague, Czech Republic; 4 Department of Comparative Biomedical Sciences, School of Veterinary Medicine, University of Surrey, Guildford, UK; 5 Department of Parasitology and Invasive Diseases, National Veterinary Research Institute in Pulawy, Pulawy, Poland; 6 Medizinisches Versorgungszentrum Labor 28 GmbH (MVZ Labor 28 GmbH), Mecklenburgische Str. 28, 14197 Berlin, Germany; 7 Institute of Microbiology, Infectious Diseases and Immunology, Charité-University Medicine Berlin, Campus Benjamin Franklin, Hindenburgdamm 30, 12200 Berlin, Germany; 8 National Veterinary Institute, Uppsala, Sweden; 9 Department of Medical Biochemistry and Microbiology, Uppsala University, Uppsala, Sweden

**Keywords:** Molecular epidemiology, Zoonotic transmission, Source tracing, MLST, Outbreak

## Abstract

**Background:**

The flagellated parasite *Giardia duodenalis* is a major and global cause of diarrhoeal disease. Eight genetically very distinct groups, known as assemblages A to H, have been recognized in the *G. duodenalis* species complex, two of which (assemblages A and B) infect humans and other mammalian hosts. Informative typing schemes are essential to understand transmission pathways, characterize outbreaks and trace zoonotic transmission. In this study, we evaluated a published multi-locus sequence typing (MLST) scheme for *G. duodenalis* assemblage A, which is based on six polymorphic markers.

**Methods:**

We genotyped 60 human-derived and 11 animal-derived *G. duodenalis* isolates collected in Europe and on other continents based on the published protocol. After retrieving previously published genotyping data and excluding isolates whose sequences showed allelic sequence heterozygosity, we analysed a dataset comprising 146 isolates.

**Results:**

We identified novel variants at five of the six markers and identified 78 distinct MLST types in the overall dataset. Phylogenetic interpretation of typing data confirmed that sub-assemblage AII only comprises human-derived isolates, whereas sub-assemblage AI comprises all animal-derived isolates and a few human-derived isolates, suggesting limited zoonotic transmission. Within sub-assemblage AII, isolates from two outbreaks, which occurred in Sweden and Italy, respectively, had unique and distinct MLST types. Population genetic analysis showed a lack of clustering by geographical origin of the isolates.

**Conclusion:**

The MLST scheme evaluated provides sufficient discriminatory power for epidemiological studies of *G. duodenalis* assemblage A.

**Graphical Abstract:**

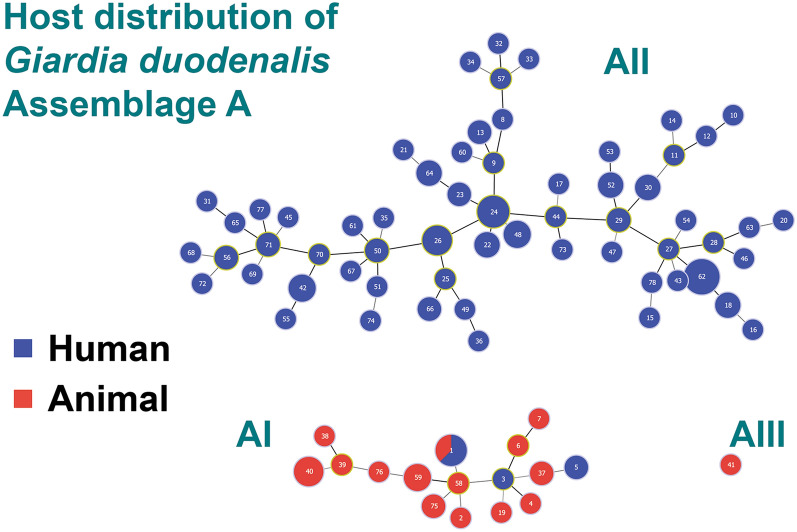

**Supplementary Information:**

The online version contains supplementary material available at 10.1186/s13071-022-05615-x.

## Background

*Giardia duodenalis* (syn., *G. intestinalis*, *G. lamblia*) is a ubiquitous flagellated protozoan that infects the upper intestinal tract of humans and other mammals [1, 2]. The parasite has a simple life-cycle consisting of an actively replicating stage that causes the symptoms, the trophozoite and a dormant and infective stage, i.e. the cyst, which is shed with host faeces and can contaminate the environment. *Giardia duodenalis* has a global distribution and causes 250–300 million symptomatic human infections annually, with a more pronounced impact in low- and middle-income countries, where the infection is usually associated with poor socioeconomic conditions [2].

In humans, the disease, called giardiasis, manifests as acute diarrhoea that can develop to a chronic stage, but the majority of infections remain asymptomatic [3]. Children and immunocompromised individuals are the most affected communities [4, 5]. In children, detrimental effects on growth, nutrition and cognitive functions have been reported [4, 6]. *Giardia duodenalis* causes disease not only in humans but also in companion animals and livestock, causing economic losses due to poor growth, weight loss and reduced productivity [7, 8].

The large body of work that has investigated protein and DNA polymorphisms in *G. duodenalis* has revealed that it is a species complex, whose members, despite being morphologically indistinguishable, can be classified into eight groups, or assemblages, separated by large genetic distances [9]. These assemblages have different host distributions, with assemblages A and B found in humans and other mammals, assemblages C and D found in dogs and other canids, assemblage E found in hoofed animals including livestock, assemblage F found in cats, assemblage G found in rodents and assemblage H found in sea mammals [10].

Additional genetic variability within assemblages has been found, mostly by applying a multi-locus sequence typing scheme (MLST) based on the beta-giardin (*bg*), triose phosphate isomerase (*tpi*) and glutamate dehydrogenase (*gdh*) genes [11, 12]. This scheme has been useful to identify sub-assemblages and genotypes, yet it offers a limited resolution due to low levels of polymorphism in these genes [7]. In 2018, Ankarklev et al. developed a novel MLST scheme for *G. duodenalis* assemblage A based on six polymorphic genomic loci, and demonstrated a large increase in discriminatory power compared to the conventional scheme [13].

Here, we have applied this new MLST scheme to a collection of isolates from Europe and other continents, mostly of human origin, and further evaluated its discriminatory power for epidemiological purposes.

## Methods

### Parasite isolates

The human isolates comprised five stool samples that were typed as assemblage A and that represented sporadic cases identified during an investigation of an outbreak of giardiasis caused by assemblage B in Italy in 2019 [14], 15 stool samples collected during another outbreak, caused by assemblage A, in Italy in 2017, and nine stool samples from unrelated cases of giardiasis in Germany. Animal isolates comprised eight faecal samples from calves collected from different provinces in Poland. Additional *G. duodenalis* isolates from humans (*n* = 31) and animals (*n* = 3; from a cat, a pig and a guinea pig, respectively) were from an archive of axenic trophozoite cultures maintained at the Charles University in Prague, Czech Republic and the Istituto Superiore di Sanità in Rome, Italy. For these axenic culture samples, the MLST marker sequences were extracted from whole genome sequence data (see following text).

Previously published genotyping data were also included in the analysis (61 datasets from [13], and 21 datasets from [15]). Isolates with sequences showing allelic sequence heterozygosity (ASH) were not retrieved from the published datasets due to possible underlying mixed infections. Therefore, six isolates from Ankarklev et al. [13] and four isolates from Woschke et al. [15] were excluded. The list of isolates is presented in Additional file 1: Table S1.

### Molecular typing

Molecular procedures were performed according to established standardized methods. Briefly for calf faeces, DNA was extracted from approximately 0.1 g (100 μl) of faecal material using the method of Millar et al. [16], with slight modifications. The temperature of thawing during repeated freeze–thawing cycles was changed to 65 °C and the number of cycles was extended to 15. DNA extracts were further purified using the GeneMATRIX PCR/DNA Clean-Up Purification Kit (EURx Ltd., Gdańsk, Poland) according to the manufacturer's instructions. DNA from the remaining faecal samples was extracted from approximately 0.2 g of faeces using the FastPrep apparatus and FastDNA Kit for soil (MP Biomedicals, LLC, Irvine, CA, USA) or the QIAamp Fast DNA Stool Mini Kit (Qiagen, Hilden, Germany) according to the respective manufacturer´s instructions. Samples were typed as *G. duodenalis* assemblage A by standard PCR methods targeting the *bg*, *tpi* or *gdh* genes [17].

Nested PCR reactions were performed on each marker as previously described [13]. Briefly, 2–5 µl of DNA was used in a 50-µl reaction volume containing 2 mM MgCl_2_, 200 µM of each deoxynucleotide triphosphate, 0.4 µM of each primer and 1.25 U of Taq DNA polymerase. All products were sequenced, on both strands, by either a commercial service (Eurofins Genomics, Germany or Genewwiz, United Kingdom) or at in-house sequencing facilities (Robert Koch-Institute) using the inner primers. Chromatograms were trimmed and analysed using the software SeqMan v. 10 (DNASTAR, Madison, WI, USA) or Geneious Prime (Biomatters Ltd., Auckland, New Zealand).

### Retrieval of sequence information from whole genome sequence data

Consensus sequences of the six markers were retrieved from a collection of 34 whole genome sequence datasets previously generated at the Department of Infectious Diseases, Istituto Superiore di Sanità, Italy, using next generation sequencing (see Additional file 1: Table S1). Bioinformatics analyses were performed using the CLC Genomics Workbench software version 10.0./11.0.1 (Qiagen), largely as previously described [18]. In brief, raw sequences from each isolate were trimmed and mapped against the *G. duodenalis* WB reference genome (sub-assemblage AI). Next, forward and reverse primers from [13] were used to identify the corresponding sequences of the six marker genes, using the tool "find binding sites and create fragments" with default settings. The consensus sequence of each gene fragment from each isolate was then extracted as a text file. The mapping results were checked for evidence of ASH at each variable position and in each gene marker in order to exclude potentially mixed isolates.

### Phylogenetic and cluster analyses

A concatenated sequence of the six markers (3414 bp) was used to infer a phylogenetic interpretation based on 452 variable positions. Trees were inferred using maximum likelihood as implemented in the MEGA X software version 10 [19]. The reliability of the clusters was evaluated using the bootstrap method with 1000 replicates. Minimum spanning trees (MST) were generated using PhyloViz [20].

### Data availability

New sequence variants from the different gene markers were deposited in GenBank under accession numbers OP450944-OP450948.

## Results

### Application of the MLST scheme to new assemblage a isolates

First, we performed PCR at the six polymorphic markers using *G. duodenalis* assemblage A genomic DNA from 20 human-derived isolates from Italy (5 sporadic cases and 15 cases from an outbreak), nine human-derived isolates from Germany and eight calf-derived isolates from Poland. All reactions yielded the expected amplification product at each marker and all products were successfully sequenced from both strands, with the exception of marker NEK15411, for which amplification failed in a single calf-derived isolate from Poland. Additionally, we retrieved the sequences of the six markers from 34 whole genome sequence datasets (see Methods section), which were obtained from cultured *G. duodenalis* isolates of human origin (*n* = 31) and animal (*n* = 3; from a cat, a pig and a guinea pig) origin. Inspections of the mapping results revealed ASH in one or more markers for six of the 34 isolates (Additional file 1: Table S1), which were therefore excluded from downstream analyses. One isolate from Germany showed ASH in one marker sequence and was therefore also excluded. After retrieving the corresponding sequences from two published studies (see Methods section), a final dataset comprising 146 isolates, of which 119 were of human and 27 of animal origin, was obtained (Additional file 1: Table S1).

Compared to the sequences obtained from the two published studies, we identified one new sequence variant at each of five markers: *CID1, HCMP6372*, *RHP26*, *DIS3* and *HCMP22547* (Additional file 2: Table S2); no additional variants were observed for marker *NEK15411*. The number of polymorphic sites at each marker (Table 1) did not vary substantially compared to previously published results, suggesting that a large fraction of the existing genetic variability at these markers has been sampled [13, 15]. Moreover, the analysis confirmed that a higher level of polymorphism did not coincide with a greater number of genotypes (Table 1; Additional file 1: Table S1). We found 78 different MLST types among the 146 isolates, compared to 42 in the study of Ankarklev et al. [13], which analysed 61 isolates, and 57 in the study of Woschke et al. [15], which included 21 additional human-derived isolates.

**Table 1:** Genetic variability at the six loci under study

Gene	Fragment size (bp) used for genotyping	Number of polymorphic sites	Number of genotypes	Number of genotypes/polymorphism
*CID1*	534	29 (28)^a^	11 (9)^b^	0.48 (0.32)^c^
*RHP26*	513	25 (23)	11 (8)	0.44 (0.35)
*HCMP22547*	555	60 (58)	12 (9)	0.2 (0.16)
*HCMP6372*	564	60 (54)	10 (7)	0.2 (0.13)
*DIS3*	615	23 (22)	10 (7)	0.45 (0.32)
*NEK15411*	633	55 (55)	13 (10)	0.23 (0.18)

Isolate SweCat171 (Assemblage AIII) was excluded from this analysis, and the number of variable sites decreased from 452 to 252

^a^Number in parentheses is the data in [13]

^b^Number in parentheses is the data in [13]

^c^Number in parentheses is the data in [13]

### Phylogenetic and cluster analyses

**Fig. 1:**
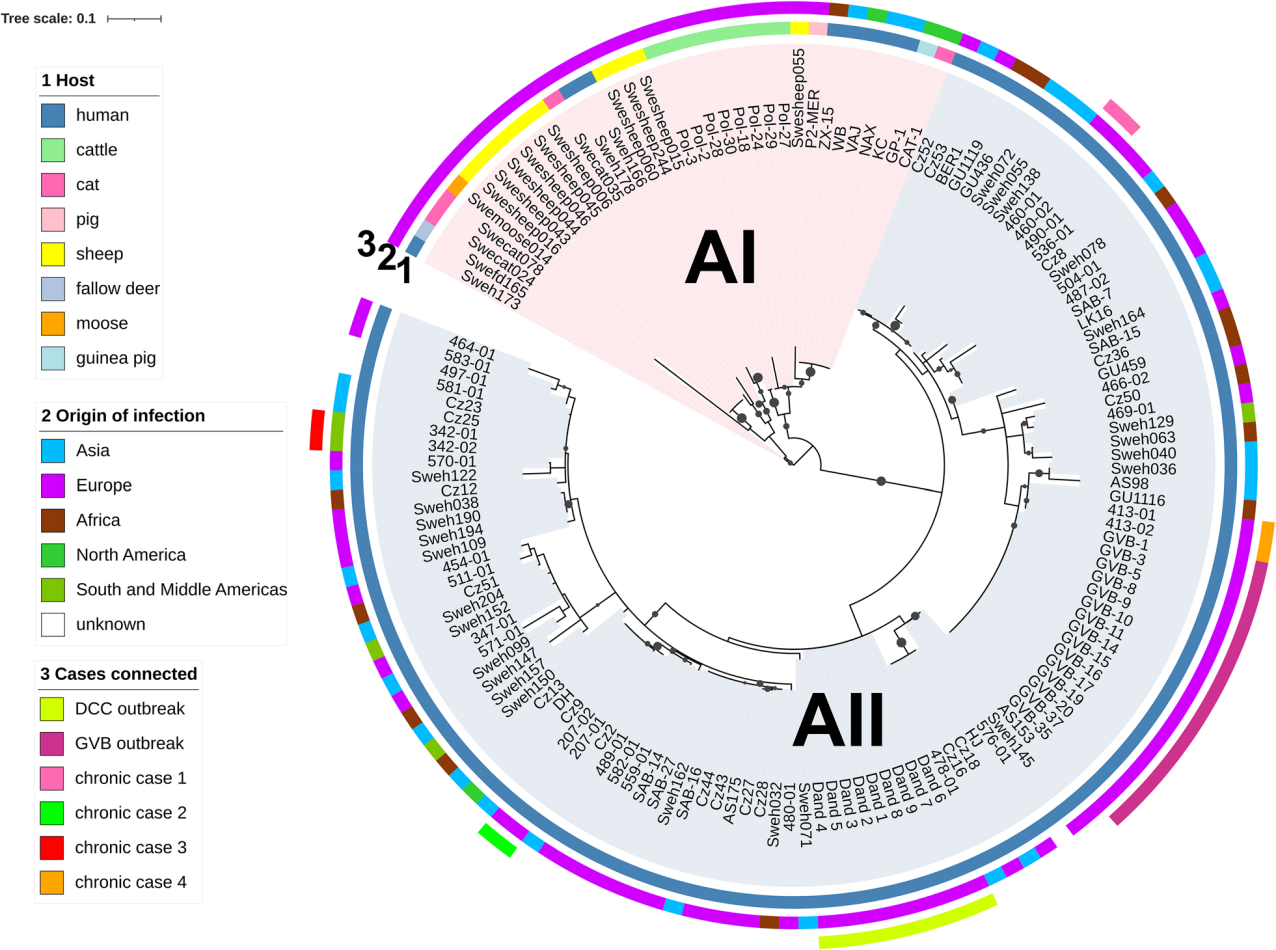
DCC outbreak in Sweden, described by Ankarklev et al. [13]; GVB outbreak in Italy, described by Resi et al. [14]; and several longitudinal samples of different patients showing similar genotypes, described by Woschke et al. [15]

The sequences of the six markers were concatenated to create a multiple alignment including all 146 isolates. A maximum likelihood tree, constructed using 452 variable positions, showed the presence of two main clusters, corresponding to sub-assemblage AI and AII (Fig. 1), with the single Assemblage AIII isolate, Swecat0171, as an outgroup (not shown). As no additional AIII samples were identified, the Swecat0171 isolate was not included in further analysis.

The sub-assemblage AI cluster comprised 34 isolates, among which 16 MLST types were identified. All animal isolates and a few human isolates clustered together. However, only one MLST type (001) is shared between humans and animals, and this type characterizes the eight in vitro-cultured sub-assemblage AI strains (Additional file 1: Table S1). The faecal-derived isolates were more variable than the axenic cultures, with 15 MLST identified in 26 isolates from various animal hosts (Additional file 1: Table S1).

The sub-assemblage AII cluster comprised 111 isolates, among which 61 MLST types were found. Of note, the 15 isolates from an Italian outbreak grouped together and shared a unique MLST type, as was the case for the nine isolates from a Swedish outbreak (Fig. 1). Additionally, longitudinal samples from four chronic giardiasis cases each also grouped together, as expected [15]. In the remaining 79 isolates, which lacked any evident epidemiological link, as many as 58 different MLST types were identified (Additional file 1: Table S1).

Notably, the clustering of the isolates within each sub-assemblage was not influenced by their geographical origin, as isolates from Europe, Africa, Asia and America are intermixed in the tree (Fig. 1). Further, analysis of MLST profiles and representation by MST showed that assemblage AII MLST types were distinct from assemblage AI MLST types, supporting the results from the phylogenetic analysis (Additional file 3: Fig. S1). Noteworthy, some identical MLST types between apparently unrelated cases (indicated by different origin of infection) were also detected (Additional file 1: Table S1; Additional file 3: Fig. S1), suggesting that most of the sequence variation of the chosen marker was already represented within the dataset, or that dominant MLST types exist. The latter is evident from Table 2, which represents the distribution of sequence types of each marker gene within the MLST types found in the present study (see also Additional file 1: Table S1). As indicated by the MST analysis, most of the sequence types of the markers were not shared between assemblage AI and AII. Strikingly, all assemblage AI isolates displayed an identical sequence at the Dis3 marker gene.

**Table 2:** Distribution of sequence types of each marker gene for the 78 different multi-locus sequence types

ST	*CID1*	*Rhp26p*	*MCMP22547*	*HCMP6372*	*Dis3*	*NEK15411*
1	8	6	3	2	16	7
2	13	9	4	8	2	14
3	6	2	21	36	2	10
4	5	28	36	13	5	33
5	17	1	3	5	4	2
6	15	28	2	1	11	1
7	6	1	2	1	35	4
8	1	1	1	1	1	3
9	5	1	3	7	1	1
10	1	0	1	4	1	1
11	1	1	1	0	0	1
12	0	0	1	0	0	0
13	0	0	0	0	0	1

The accession numbers and references of sequence types can be found in Additional file 2: Table S2

*ST* Sequence type

## Discussion

Traditionally, molecular typing of *G. duodenalis* isolates has been largely based on sequence analyses of three marker genes, namely those encoding for beta-giardin (BG), triose phosphate isomerase (TPI) and glutamate dehydrogenase (GDH) [11, 12]. This typing scheme allows the discrimination of different assemblages and sub-assemblages of the parasite, but lacks the resolution required for epidemiological applications, such as tracing of outbreaks and zoonotic transmission.

In 2018, Ankarklev et al. published a novel MLST scheme for *G. duodenalis* assemblage A, one of the two assemblages infecting humans [13]. The scheme, based on six highly polymorphic genes located on different chromosomes, demonstrated good resolution, with 42 different MLST types identified in 61 isolates. The authors demonstrated the applicability of the scheme to infer zoonotic transmission and to support outbreak investigation [13]. More recently, Woschke et al. applied this typing scheme to human isolates from Germany and identified 15 novel MLST types [15]. Importantly, the authors reported the presence of an identical isolate type in samples collected longitudinally from patients with chronic infection.

In the present study, we aimed at providing additional data from isolates of human and animal origin representing infections acquired in additional European countries or on other continents. Sequencing of the six markers (*CID1, HCMP6372*, *RHP26*, *DIS3*, *HCMP22547*, *NEK15411*) from 65 isolates identified only five novel sequence variants at five of the six markers; this slight increase suggests that a large fraction of the existing variability has been sampled. On the other hand, the number of different MLST increased from 57 [15] to 78, showing additional combinations of sequence types.

Zoonotic transmission of *G. duodenalis* has been an open research question for many years [7, 17, 21]. A recent review reassessed the prevalence and distribution of genotypes in animals, based on the *bg*, *tpi* and *gdh* loci [8]. The authors confirmed that host-adapted assemblages (C to G) are largely more prevalent than the potentially zoonotic assemblages A and B, and that sub-assemblage AI is the dominant type in both livestock and companion animals infected with *G. duodenalis* assemblage A [8]. This latter finding is confirmed by our data, as all animal isolates belonged to sub-assemblage AI, with the exception of a cat isolate of sub-assemblage AIII. Only eight human isolates clustered with sub-assemblage AI, and only a single MLST type was shared between humans and animals. These results suggest low rates of zoonotic transmission, although well-designed epidemiologic studies, particularly in areas where close contact between humans and animals is common, are needed to draw robust conclusions.

*Giardia* is a major cause of waterborne [22] and foodborne [23] outbreaks of enteric disease in industrialized nations, and genotyping strains in outbreak situations is of epidemiological relevance. In this study, we show that 15 isolates from an outbreak in Italy all shared the same MLST, which was not found in any other isolate included in this study. It is noteworthy that isolates from a Swedish outbreak [13] also shared an identical MLST type, yet distinct from that characterizing the Italian outbreak. Besides outbreak investigation, the MLST may help to attribute the main source of human assemblage A infections in endemic countries, and elucidate the proportion of person-to-person transmission versus infection via food/water in specific local settings.

The population structure of *G. duodenalis* at the assemblage level is not clear, in part because the commonly used *bg*, *tpi* and *gdh* markers are too conserved. The new typing scheme for assemblage A [13] is based on six polymorphic single-copy genes identified by whole-genome comparison of just three assemblage A isolates, namely WB (AI), AS98 and AS175 (both AII). Therefore, broader genomic sequence variation within assemblage A isolates likely exists [13, 15]. In comparison to sub-assemblage AI, which is found in animals and humans, sub-assemblage AII is only found in humans and shows a larger genetic variability within the analysed dataset. However, it should be noted that this observation might be biased by the fact that the MLST type of all in vitro-cultured sub-assemblage AI strains was identical, whereas the AI isolates typed from faeces were more diverse. As these isolates had no obvious epidemiological connection, this indicates that the axenization of assemblage AI isolates may introduce a bias and select specific variants. Whether this is indeed the case and the identity of the underlying biological mechanisms need to be addressed in future studies using larger and more defined datasets.

Genotyping of *G. duodenalis* from faecal samples by classical MLST methods, as shown in the present study, poses several potential issues. First, due to the tetraploid nature of the genome [1], heterozygote organisms can occur, from which blurred typing results are generated due to ASH. It has been reported that the occurrence of ASH is lower in sub-assemblage A than in assemblage B [10, 13, 15], and indeed in most assemblage A isolates no ASH was detected [10, 13, 15]. This indicates that assemblage A isolates are mainly homozygotic organisms. However, it should be noted that a minor fraction of assemblage AII isolates have heterozygote nucleotides in some marker sequences [10, 13, 15]. Second, it is important to note that the majority of the samples included in the present study are clinical faecal samples from either infected humans or animals. An infection originates from a population of parasites that potentially may have different MLST types. It is also been suggested that recombination occurs in *Giardia* [13], which also may add to the occurrence of several MLST types in a single sample. Hence, whether observed variability within a sample is due to true heterozygotes, populations or to mixed infections in the faecal sample is not known and we excluded those isolates with unclear genotypes from the analysed dataset.

## Conclusions

We further evaluated the novel MLST scheme for assemblage A proposed by Ankarklev et al. [13] on a larger and more geographically spread dataset. We confirm that typing resolution is sufficient for epidemiological purposes, but also identified identical MLST types in unrelated samples, which emphasizes the importance of contextualizing the data during their interpretation. Additional collection of informative datasets, as shown in the present work, will continually improve the usability of this new typing scheme.

## Supplementary Information

**Additional file 1: Table S1.** List of isolates and MLST typing data.

**Additional file 3: Figure S1.** Sub-assemblage AI- and AII-specific minimum spanning tree.

**Additional file 2: Table S2.** Isolate ID and accession numbers of all sequence types of individual markers.

## Data Availability

New sequence variants were deposited in GenBank under accession numbers OP450944-OP450948.

## Abbreviations

ASH: Allelic sequence heterozygosity
*bg*: Beta-giardin gene
*gdh*: Glutamate dehydrogenase gene
MLST: Multi-locus sequence typing
MST: Minimum spanning tree
*tpi*: Triose phosphate isomerase gene
