# Supplementary material for: Extensive testing of a multi-locus sequence typing scheme for Giardia duodenalis assemblage A confirms its good discriminatory power
Source: Parasit Vectors. 2022 Dec 26;15:489. doi: 10.1186/s13071-022-05615-x (PMC9791779; doi:10.1186/s13071-022-05615-x)
Supplement: Supplementary file 2 — Additional file 3: Figure S1. Sub-assemblage AI- and AII-specific minimum spanning tree. [file 13071_2022_5615_MOESM2_ESM.docx]

**Table S2**: Isolate ID and accession numbers of all sequence types described in Ankarklev et al. (2018) (grey), Woschke et al. (2021) (blue) and the present study (yellow).

|  | **Gene** | | | | | |
| --- | --- | --- | --- | --- | --- | --- |
| Sequence code | **CID1** | **Rhp26p** | **HCMP22547** | **HCMP6372** | **Dis3** | **NEK15411** |
|  | Isolate/acc no | Isolate/acc no | Isolate/acc no | Isolate/acc no | Isolate/acc no | Isolate/acc no |
| 1 | WB | WB | WB | WB | WB | WB |
|  | MG520215 | MG520254 | MG520233 | MG520225 | MG520263 | MG520243 |
| 2 | Sweh038 | Sweh173 | Sweh173 | Sweh166 | Sweh072 | AS98 |
|  | MG520216 | MG520255 | MG520234 | MG520226 | MG520264 | MG520244 |
| 3 | AS98 | AS98 | AS98 | AS98 | AS98 | Sweh040 |
|  | MG520217 | MG520256 | MG520235 | MG520227 | MG520265 | MG520245 |
| 4 | GU1116 | AS175 | AS175 | AS175 | Sweh036 | AS175 |
|  | MG520218 | MG520257 | MG520236 | MG520228 | MG520266 | MG520246 |
| 5 | AS175 | Sweh152 | Sweh166 | Sweh063 | AS153 | Sweh038 |
|  | MG520219 | MG520258 | MG520237 | MG520229 | MG520267 | MG520247 |
| 6 | Sweh099 | Sweh038 | Sweh078 | Swemoose014 | Sweh038 | Sweh204 |
|  | MG520220 | MG520259 | MG520238 | MG520230 | MG520268 | MG520248 |
| 7 | Sweh071 | Swemoose014 | Swesheep006 | Swecat078 | AS175 | Swemoose014 |
|  | MG520221 | MG520260 | MG520239 | MG520231 | MG520269 | MG520249 |
| 8 | Swesheep015 | Swecat078 | Swecat078 | Swecat171 | Swecat171 | Sweh166 |
|  | MG520222 | MG520261 | MG520240 | MG520232 | MG520270 | MG520250 |
| 9 | Swemoose014 | Swecat171 | Swecat035 | 464-01 | 464-01 | Swesheep060 |
|  | MG520223 | MG520262 | MG520241 | MT879095 | MT879093 | MG520251 |
| 10 | Swecat171 | 207-01 | Swecat171 | Pol-18 | Cz51 | Swecat078 |
|  | MG520224 | MT879098 | MG520242 | OP450947 | OP450948 | MG520252 |
| 11 | 581-01 | Cz27 | 466-02/ |  |  | Swecat171 |
|  | OP450944 | OP450945 | MT879094 |  |  | MG520253 |
| 12 |  |  | 559-01 |  |  | 453-02 |
|  |  |  | OP450946 |  |  | MT879097 |
| 13 |  |  |  |  |  | 511-01 |
|  |  |  |  |  |  | MT879096 |
