# Supplementary material for: Extensive testing of a multi-locus sequence typing scheme for Giardia duodenalis assemblage A confirms its good discriminatory power
Source: Parasit Vectors. 2022 Dec 26;15:489. doi: 10.1186/s13071-022-05615-x (PMC9791779; doi:10.1186/s13071-022-05615-x)
Supplement: Supplementary file 3 — Additional file 2: Table S2. Isolate ID and accession numbers of all sequence types of individual markers. [file 13071_2022_5615_MOESM3_ESM.pdf]

Additional file 2

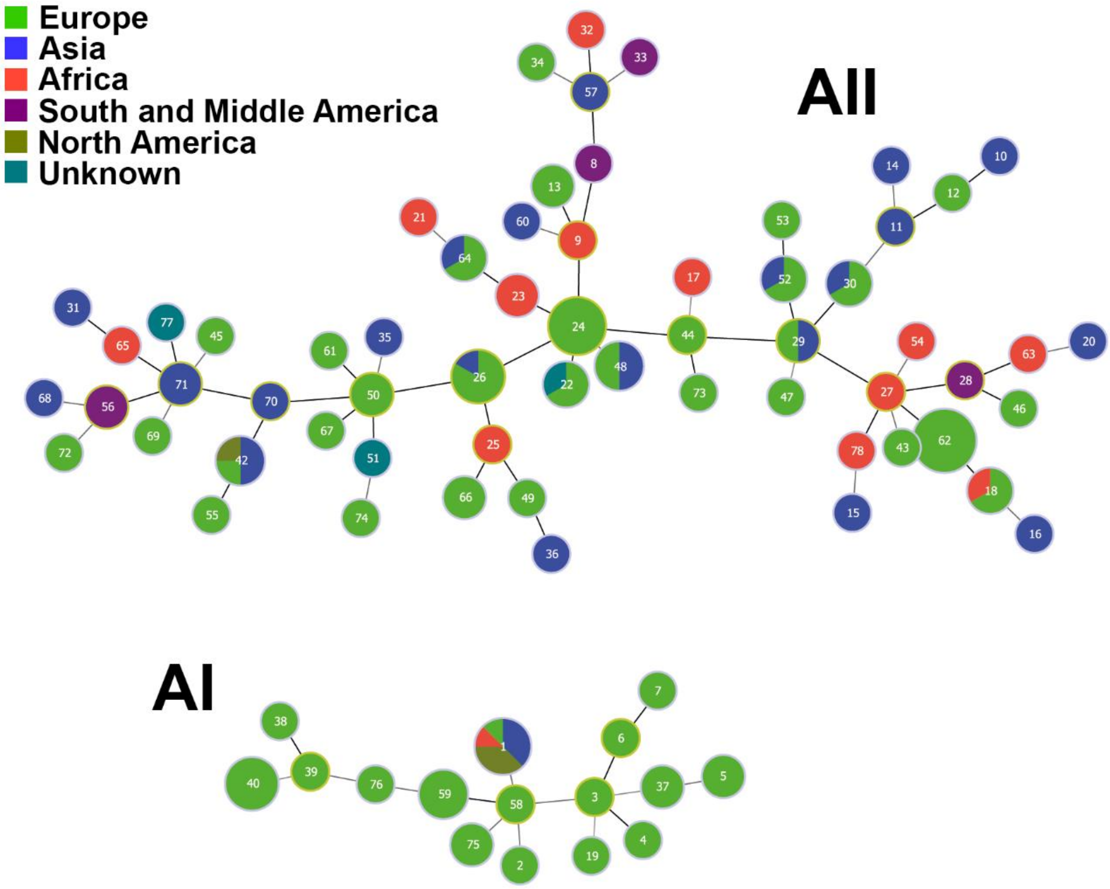

Fig. S1. Sub-assemblage AI- and AII-specific minimum spanning tree (MST). MST analysis based on profile data of all 77 various MLST types (except the single assemblage AIII MLST). Each spot represents one MLST type and size indicates the number of underlying samples (in log scale). Suspected origins of isolates are indicated by the color code.
